# Supplementary figures and images for: Redefining the ancestral origins of the interleukin-1 superfamily
Source: Nat Commun. 2018 Mar 20;9:1156. doi: 10.1038/s41467-018-03362-1 (PMC5861070; doi:10.1038/s41467-018-03362-1)

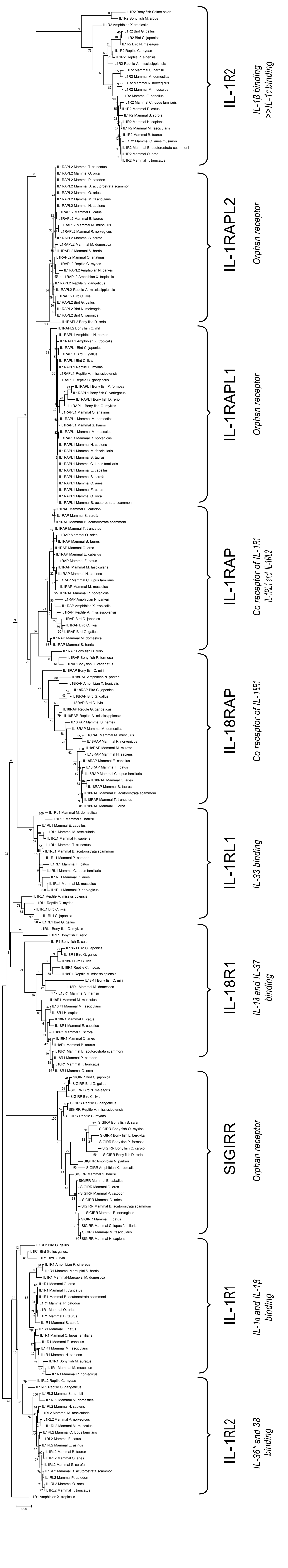

Supplement: Supplementary file 2 — Supplementary Data 1(PDF 496 kb) [file 41467_2018_3362_MOESM2_ESM.pdf]

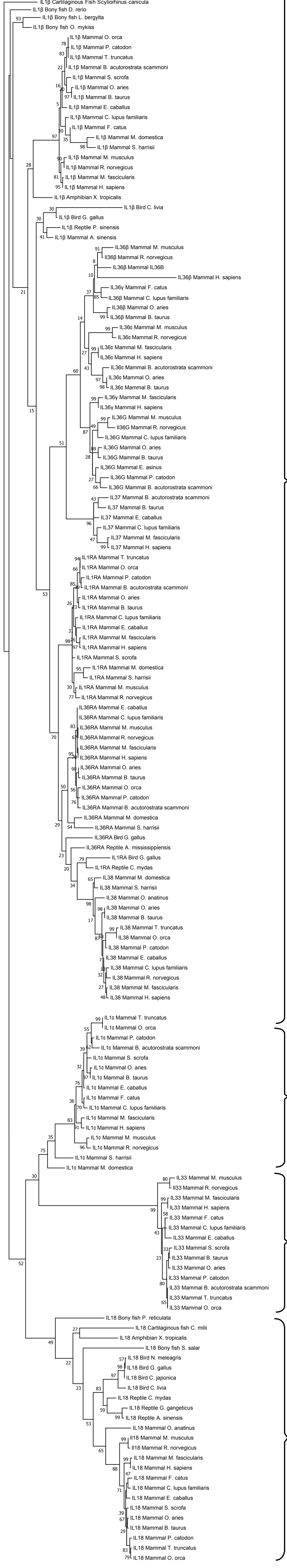

IL-1β, IL-1RA, IL-36\*, IL-37, IL-38 Cluster

IL-1α

IL-33

IL-18

Supplement: Supplementary file 3 — Supplementary Data 2(PDF 582 kb) [file 41467_2018_3362_MOESM3_ESM.pdf]

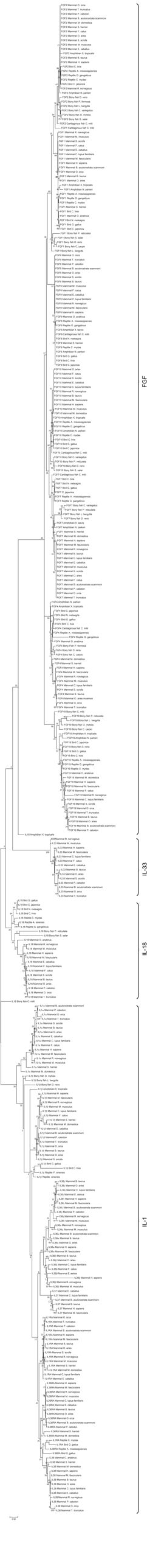

Supplement: Supplementary file 4 — Supplementary Data 3(PDF 705 kb) [file 41467_2018_3362_MOESM4_ESM.pdf]

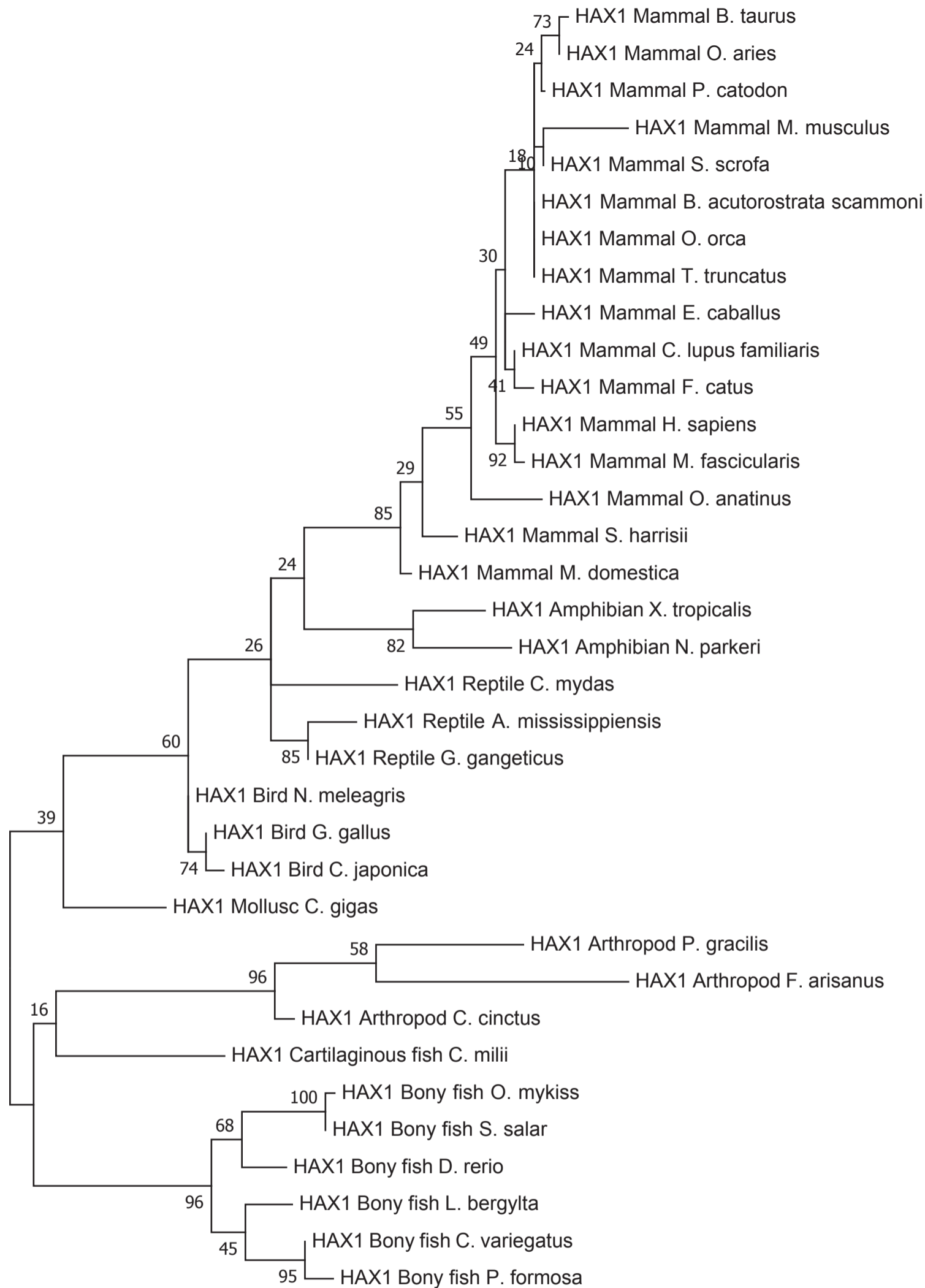

0.20

Supplement: Supplementary file 5 — Supplementary Data 4(PDF 206 kb) [file 41467_2018_3362_MOESM5_ESM.pdf]

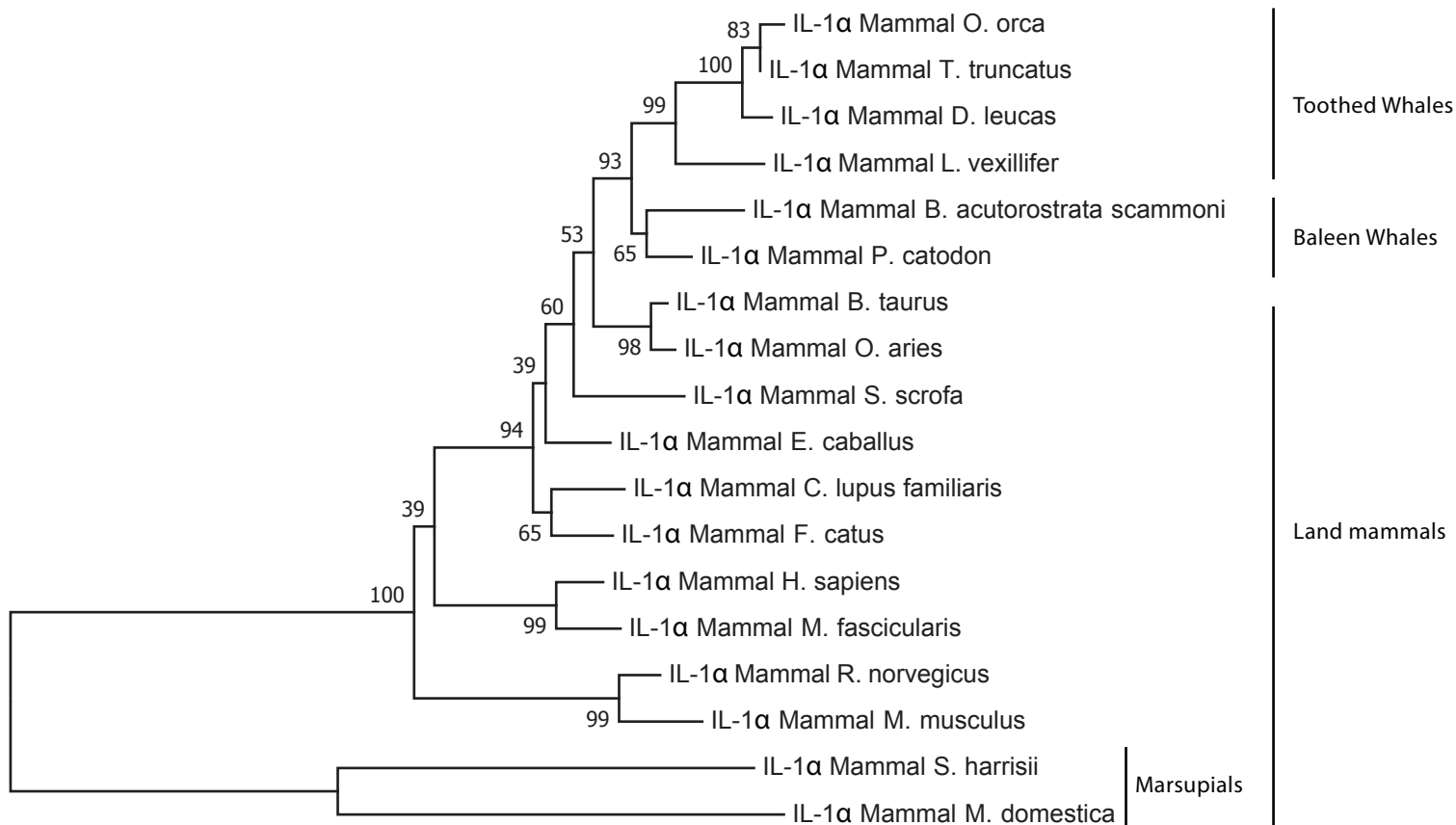

0.10

Supplement: Supplementary file 6 — Supplementary Data 5(PDF 224 kb) [file 41467_2018_3362_MOESM6_ESM.pdf]

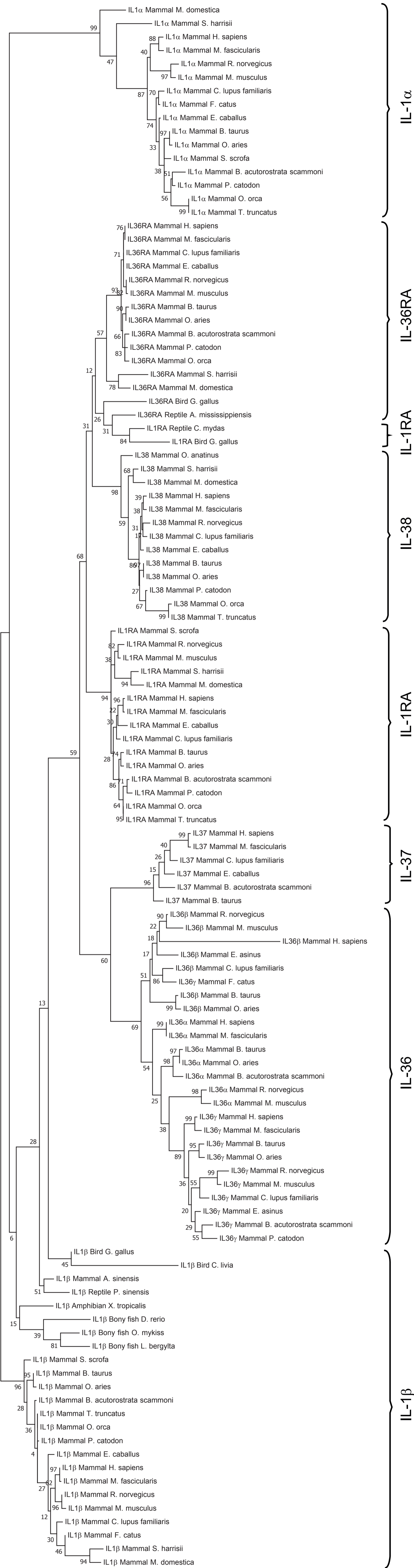

0.20

Supplement: Supplementary file 7 — Supplementary Data 6(PDF 260 kb) [file 41467_2018_3362_MOESM7_ESM.pdf]

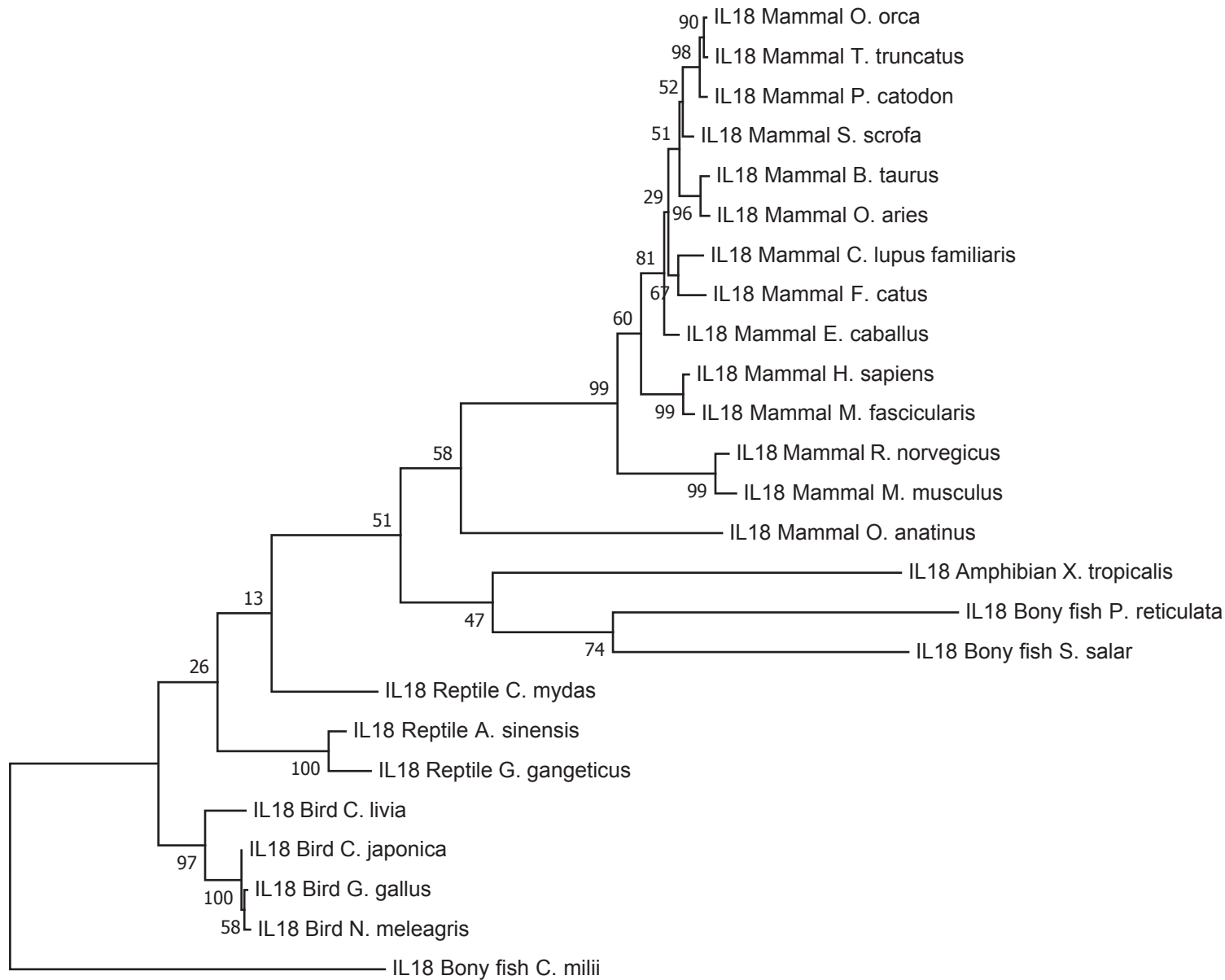

0.20

Supplement: Supplementary file 8 — Supplementary Data 7(PDF 183 kb) [file 41467_2018_3362_MOESM8_ESM.pdf]

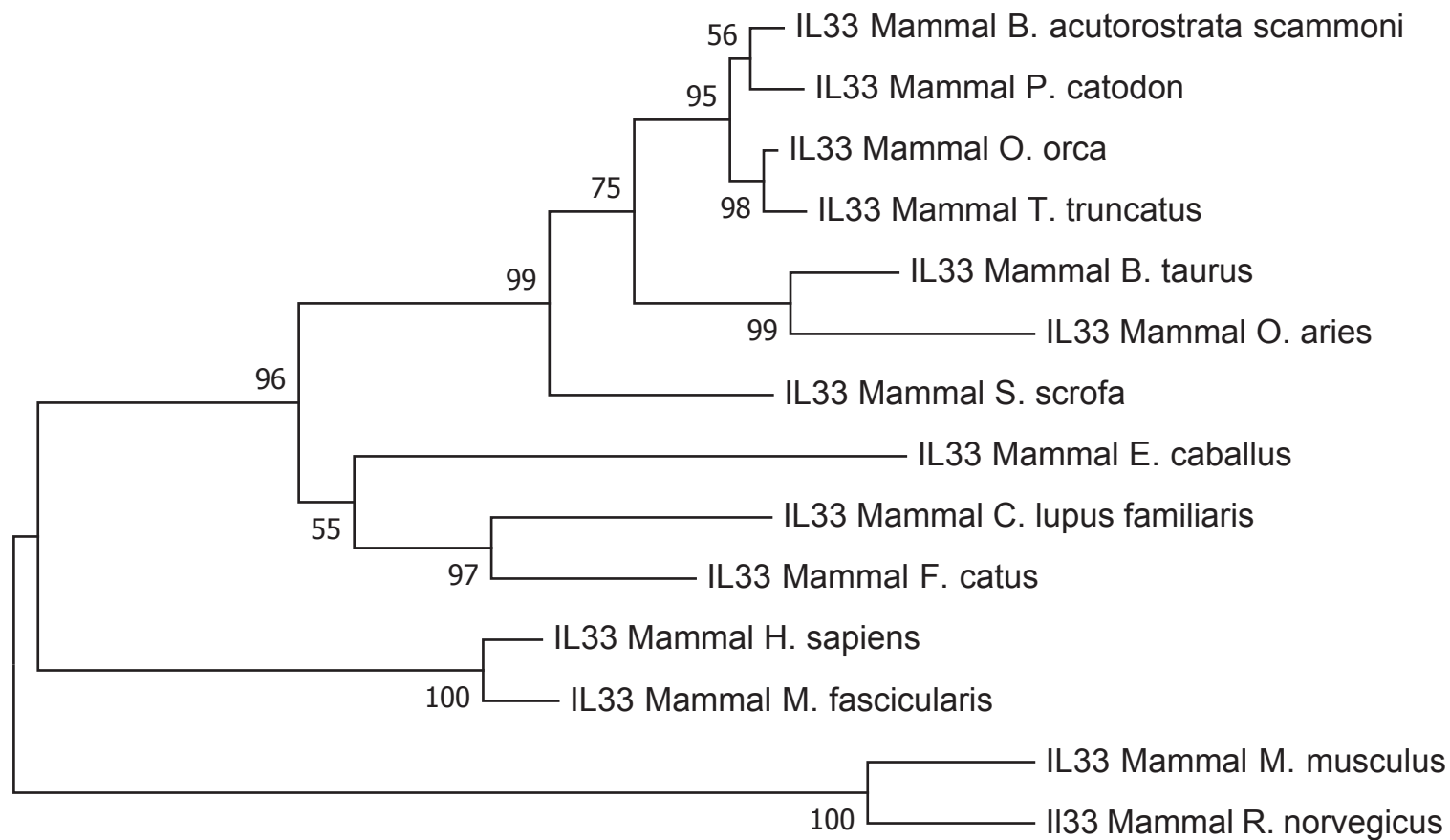

0.050

Supplement: Supplementary file 9 — Supplementary Data 8(PDF 178 kb) [file 41467_2018_3362_MOESM9_ESM.pdf]
