# Supplementary material for: Redefining the ancestral origins of the interleukin-1 superfamily
Source: Nat Commun. 2018 Mar 20;9:1156. doi: 10.1038/s41467-018-03362-1 (PMC5861070; doi:10.1038/s41467-018-03362-1)
Supplement: Supplementary file 10 — Supplementary Data 9(PDF 387 kb) [file 41467_2018_3362_MOESM10_ESM.pdf]

Taxa	Gene ID	RefSeq status	Clade
**IL1A**			
Homo sapien	147760	Reviewed	Mammal
Macaca fascicularis	101926546	Provisional	Mammal
Bos taurus	281250	Provisional	Mammal
Rattus norvegicus	24493	Provisional	Mammal
Canis lupus familiaris	403782	Provisional	Mammal
Equus caballus	100064969	Validated	Mammal
Mus musculus	16175	Model	Mammal
Sus scrofa	397094	Model	Mammal
Ovis Aries	443404	Provisional	Mammal
Felis catus	493944	Provisional	Mammal
Ornithorhynchus anatinus	NA		Mammal
Sarcophilus harrisii	105749132	Model	Mammal
Monodelphis domestica	103105940	Model	Mammal
Balaenoptera acutorostrata scammoni	103008155	Model	Mammal
Physeter catodon	102993910	Model	Mammal
Orcinus orca	101272616	Model	Mammal
Tursiops truncatus	101319101	Model	Mammal
Delphinapterus leucas	11187501	Model	Mammal
Alligator mississippiensis	NA		Reptile
Gavialis gangeticus	NA		Reptile
Chelonia mydas	NA		Reptile
Xenopus tropicalis	NA		Amphibian
Nanorana parkeri	NA		Amphibian
Gallus gallus	NA		Bird
Columba ivia	NA		Bird
Coturnix japonica	NA		Bird
Numida meleagris	NA		Bird
Danio rerio	NA		Bony fish
Cyprinus carpio	NA		Bony fish
Mesocricetus auratus	NA		Bony Fish
Oncorhynchus mykiss	NA		Bony fish
Callorhinchus Milii	NA		Cartilaginous fish
Salmo salar	NA		Bony fish
Cyprinus carpio	NA		Bony Fish
Labrus bergylta	NA		Bony Fish
Scyliorhinus canicula	NA		Cartilaginous fish

**IL1B**			
Homo sapiens	3553	Reviewed	Mammal
Macaca fascicularis	102119749	Validated	Mammal
Bos taurus	281251	Provisional	Mammal
Rattus norvegicus	2891	Provisional	Mammal
Canis lupus familiaris	403974	Model	Mammal
Equus caballus	100052414	Provisional	Mammal
Mus musculus	16176	Reviewed	Mammal

Sus scrofa	397122	Provisional	Mammal
Ovis Aries	443539	Provisional	Mammal
Felis catus	768274	Provisional	Mammal
Sarcophilus harrisii	100922350	Model	Mammal
Monodelphis domestica	100026024	Model	Mammal
Balaenoptera acutorostrata scammoni	103008438	Model	Mammal
Physeter catodon	102994188	Model	Mammal
Orcinus orca	101284892	Model	Mammal
Tursiops truncatus	101318809	Model	Mammal
Alligator sinensis	102369595	Model	Reptile
Pelodiscus sinensis	102448244	Provisional	Reptile
Chelonia mydas	NA		Reptile
Xenopus tropicalis	100487038	Model	Amphibian
Nanorana parkeri	NA	Model	Amphibian
Gallus gallus	395196	Model	Bird
Columba Livia	102090585	Model	Bird
Coturnix japonica	NA	Model	Bird
Numida meleagris	NA	Model	Bird
Danio rerio	405770	Validated	Bony fish
Cyprinodon variegatus	NA		Bony fish
Poecilia formosa	NA		Bony fish
Oncorhynchus mykiss	100136024	Provisional	Bony fish
Callorhinchus Milii	NA		Cartilaginous fish
Salmo salar	NA		Bony fish
Cyprinus carpio	NA		Bony fish
Labrus bergylta	109997024	Model	Bony fish
Scyliorhinus canicula	10241835	Model	Cartilaginous fish
**IL18**			
Homo sapiens	3606	Model	Mammal
Macaca fascicularis	102135101	Model	Mammal
Bos taurus	281249	Model	Mammal
Rattus norvegicus	2889	PROVISIONAL	Mammal
Canis lupus familiaris	403796	Model	Mammal
Equus caballus	100034216	Model	Mammal
Mus musculus	16173	REVIEWED	Mammal
Sus scrofa	397057	PROVISIONAL	Mammal
Ovis Aries	443206	Model	Mammal
Felis catus	493688	Model	Mammal
Ornithorhynchus anatinus	100083773	Model	Mammal
Sarcophilus harrisii	NA	Model	Mammal
Monodelphis domestica	NA	Model	Mammal
Balaenoptera acutorostrata scammoni	NA	Model	Mammal
Physeter catodon	102976313	Model	Mammal
Orcinus orca	101271274	Model	Mammal
Tursiops truncatus	101334331	Model	Mammal
Alligator mississippiensis	102369831	Model	Reptile
Gavialis gangeticus	109292958	Model	Reptile
Chelonia mydas	102933793	Model	Reptile
Xenopus tropicalis	108648098	Model	Amphibian

Nanorana parkeri	NA	Model	Amphibian
Gallus gallus	395312	Model	Bird
Columba Livia	102093971	Model	Bird
Coturnix japonica	107324129	Model	Bird
Numida meleagris	110387565	Model	Bird
Danio rerio	NA	Model	Bony fish
Cyprinodon variegatus	NA	Model	Bony fish
Poecilia reticulata	103474370	Model	Bony fish
Oncorhynchus mykiss	NA	Model	Bony fish
Callorhinchus Milii	103191153	Model	Cartilaginous fish
Salmo salar	100196379	PROVISIONAL	Bony fish
Cyprinus carpio	NA	Model	Bony fish
Labrus bergylta	NA	Model	Bony fish
Scyliorhinus canicula	NA		Cartilaginous fish

**IL33**			
Homo sapiens	608678	Model	Mammal
Macaca fascicularis	102140695	Model	Mammal
Bos taurus	507054	Model	Mammal
Rattus norvegicus	1311155	Provisional	Mammal
Canis lupus familiaris	403810	Model	Mammal
Equus caballus	100059908	Model	Mammal
Mus musculus	77125	Validated	Mammal
Sus scrofa	100518643	Model	Mammal
Ovis Aries	101116705	Model	Mammal
Felis catus	101093403	Model	Mammal
Ornithorhynchus anatinus	NA		Mammal
Sarcophilus harrisii	NA		Mammal
Monodelphis domestica	NA		Mammal
Balaenoptera acutorostrata scammoni	103011945	Model	Mammal
Physeter catodon	102989473	Model	Mammal
Orcinus orca	101277857	Model	Mammal
Tursiops truncatus	101319264	Model	Mammal
Alligator mississippiensis	NA		Reptile
Gavialis gangeticus	NA		Reptile
Chelonia mydas	NA		Reptile
Xenopus tropicalis	NA		Amphibian
Nanorana parkeri	NA		Amphibian
Gallus gallus	NA		Bird
Columba Livia	NA		Bird
Coturnix japonica	NA		Bird
Numida meleagris	NA		Bird
Danio rerio	NA		Bony fish
Cyprinodon variegatus	NA		Bony fish
Poecilia formosa	NA		Bony fish
Oncorhynchus mykiss	NA		Bony fish
Callorhinchus Milii	NA		Cartilaginous fish
Salmo salar	NA		Bony fish
Cyprinus carpio	NA		Bony fish
Labrus bergylta	NA		Bony fish

Scyliorhinus canicula	NA		Cartilaginous fish
**IL36A**			
Homo sapiens	605509	Model	Mammal
Macaca fascicularis	102123034	Model	Mammal
Bos taurus	523429	Model	Mammal
Rattus norvegicus	1306869	Provisional	Mammal
Canis lupus familiaris	NA		Mammal
Equus caballus	NA		Mammal
Mus musculus	1859324	Provisional	Mammal
Sus scrofa	NA		Mammal
Ovis Aries	101106251	Model	Mammal
Felis catus	NA		Mammal
Ornithorhynchus anatinus	NA		Mammal
Sarcophilus harrisii	NA		Mammal
Monodelphis domestica	NA		Mammal
Balaenoptera acutorostrata scammoni	103019938	Model	Mammal
Physeter catodon	NA		Mammal
Orcinus orca	NA		Mammal
Tursiops truncatus	NA		Mammal
Alligator mississippiensis	NA		Reptile
Gavialis gangeticus	NA		Reptile
Chelonia mydas	NA		Reptile
Xenopus tropicalis	NA		Amphibian
Nanorana parkeri	NA		Amphibian
Gallus gallus	NA		Bird
Columba Livia	NA		Bird
Coturnix japonica	NA		Bird
Numida meleagris	NA		Bird
Danio rerio	NA		Bony fish
Cyprinodon variegatus	NA		Bony fish
Poecilia formosa	NA		Bony fish
Oncorhynchus mykiss	NA		Bony fish
Callorhinchus Milii	NA		Cartilaginous fish
Salmo salar	NA		Bony fish
Cyprinus carpio	NA		Bony fish
Labrus bergylta	NA		Bony fish
Scyliorhinus canicula	NA		Cartilaginous fish
**IL36B**			
Homo sapiens	27177	Reviewed	Mammal
Macaca fascicularis	NA		Mammal
Bos taurus	100297786	Model	Mammal
Rattus norvegicus	1307847	Model	Mammal
Canis lupus familiaris	483068	Model	Mammal
Equus asinus	100065096	Model	Mammal
Mus musculus	69677	Provisional	Mammal
Sus scrofa	NA		Mammal
Ovis Aries	101105996	Model	Mammal
Felis catus	NA		Mammal

Ornithorhynchus anatinus	NA		Mammal
Sarcophilus harrisii	NA		Mammal
Monodelphis domestica	NA		Mammal
Balaenoptera acutorostrata scammoni	NA		Mammal
Physeter catodon	NA		Mammal
Orcinus orca	NA		Mammal
Tursiops truncatus	NA		Mammal
Alligator mississippiensis	NA		Reptile
Gavialis gangeticus	NA		Reptile
Chelonia mydas	NA		Reptile
Xenopus tropicalis	NA		Amphibian
Nanorana parkeri	NA		Amphibian
Gallus gallus	NA		Bird
Columba Livia	NA		Bird
Coturnix japonica	NA		Bird
Numida meleagris	NA		Bird
Danio rerio	NA		Bony fish
Cyprinodon variegatus	NA		Bony fish
Poecilia formosa	NA		Bony fish
Oncorhynchus mykiss	NA		Bony fish
Callorhinchus Milii	NA		Cartilaginous fish
Salmo salar	NA		Bony fish
Cyprinus carpio	NA		Bony fish
Labrus bergylta	NA		Bony fish
Scyliorhinus canicula	NA		Cartilaginous fish
**IL36G**			
Homo sapiens	56300	Reviewed	Mammal
Macaca fascicularis	102121515	Model	Mammal
Bos taurus	615762	Model	Mammal
Rattus norvegicus	499744	Provisional	Mammal
Canis lupus familiaris	100686137	Model	Mammal
Equus asinus	106838560	Model	Mammal
Mus musculus	215257	Validated	Mammal
Sus scrofa	NA		Mammal
Ovis Aries	101106514	Model	Mammal
Felis catus	101081377	Model	Mammal
Ornithorhynchus anatinus	NA		Mammal
Sarcophilus harrisii	NA		Mammal
Monodelphis domestica	NA		Mammal
Balaenoptera acutorostrata scammoni	103019653	Model	Mammal
Physeter catodon	102979011	Model	Mammal
Orcinus orca	NA		Mammal
Tursiops truncatus	NA		Mammal
Alligator mississippiensis	NA		Reptile
Gavialis gangeticus	NA		Reptile
Chelonia mydas	NA		Reptile
Xenopus tropicalis	NA		Amphibian
Nanorana parkeri	NA		Amphibian
Gallus gallus	NA		Bird

Columba Livia	NA		Bird
Coturnix japonica	NA		Bird
Numida meleagris	NA		Bird
Danio rerio	NA		Bony fish
Cyprinodon variegatus	NA		Bony fish
Poecilia formosa	NA		Bony fish
Oncorhynchus mykiss	NA		Bony fish
Callorhinchus Milii	NA		Cartilaginous fish
Salmo salar	NA		Bony fish
Cyprinus carpio	NA		Bony fish
Labrus bergylta	NA		Bony fish
Scyliorhinus canicula	NA		Cartilaginous fish

IL37			
Homo sapiens	27178	Model	Mammal
Macaca fascicularis	102120382	Model	Mammal
Bos taurus	786493	Model	Mammal
Rattus norvegicus	NA		Mammal
Canis lupus familiaris	100686057	Model	Mammal
Equus caballus	100052470	Model	Mammal
Mus musculus	NA		Mammal
Sus scrofa	NA		Mammal
Ovis Aries	NA		Mammal
Felis catus	NA		Mammal
Ornithorhynchus anatinus	NA		Mammal
Sarcophilus harrisii	NA		Mammal
Monodelphis domestica	NA		Mammal
Balaenoptera acutorostrata scammoni	103019118	Model	Mammal
Physeter catodon	NA		Mammal
Orcinus orca	NA		Mammal
Tursiops truncatus	NA		Mammal
Alligator mississippiensis	NA		Reptile
Gavialis gangeticus	NA		Reptile
Chelonia mydas	NA		Reptile
Xenopus tropicalis	NA		Amphibian
Nanorana parkeri	NA		Amphibian
Gallus gallus	NA		Bird
Columba Livia	NA		Bird
Coturnix japonica	NA		Bird
Numida meleagris	NA		Bird
Danio rerio	NA		Bony fish
Cyprinodon variegatus	NA		Bony fish
Poecilia formosa	NA		Bony fish
Oncorhynchus mykiss	NA		Bony fish
Callorhinchus Milii	NA		Cartilaginous fish
Salmo salar	NA		Bony fish
Cyprinus carpio	NA		Bony fish
Labrus bergylta	NA		Bony fish
Scyliorhinus canicula	NA		Cartilaginous fish

IL38			
Homo sapiens	615296	Reviewed	Mammal
Macaca fascicularis	102125744	Model	Mammal
Bos taurus	615702	Provisional	Mammal
Rattus norvegicus	1305635	Model	Mammal
Canis lupus familiaris	611873	Model	Mammal
Equus caballus	100052532	Model	Mammal
Mus musculus	NA		Mammal
Sus scrofa	NA		Mammal
Ovis Aries	101111084	Model	Mammal
Felis catus	NA		Mammal
Ornithorhynchus anatinus	10092682	Model	Mammal
Sarcophilus harrisii	100919996	Model	Mammal
Monodelphis domestica	100013210	Model	Mammal
Balaenoptera acutorostrata scammoni	103009890	Model	Mammal
Physeter catodon	102976532	Model	Mammal
Orcinus orca	105748873	Model	Mammal
Tursiops truncatus	101328728	Model	Mammal
Alligator mississippiensis	NA		Reptile
Gavialis gangeticus	NA		Reptile
Chelonia mydas	NA		Reptile
Xenopus tropicalis	NA		Amphibian
Nanorana parkeri	NA		Amphibian
Gallus gallus	NA		Bird
Columba Livia	NA		Bird
Coturnix japonica	NA		Bird
Numida meleagris	NA		Bird
Danio rerio	NA		Bony fish
Cyprinodon variegatus	NA		Bony fish
Poecilia formosa	NA		Bony fish
Oncorhynchus mykiss	NA		Bony fish
Callorhinchus Milii	NA		Cartilaginous fish
Salmo salar	NA		Bony fish
Cyprinus carpio	NA		Bony fish
Labrus bergylta	NA		Bony fish
Scyliorhinus canicula	NA		Cartilaginous fish

IL1RN			
Homo sapiens	3557	Reviewed	Mammal
Macaca fascicularis	102126145	Model	Mammal
Bos taurus	281860	Model	Mammal
Rattus norvegicus	621159	Model	Mammal
Canis lupus familiaris	403660	Validated	Mammal
Equus caballus	100034236	Model	Mammal
Mus musculus	16181	Validated	Mammal
Sus scrofa	397499	Provisional	Mammal
Ovis Aries	780505	Model	Mammal
Felis catus	NA		Mammal
Ornithorhynchus anatinus	NA		Mammal
Sarcophilus harrisii	100923652	Model	Mammal

Monodelphis domestica	100026175	Model	Mammal
Balaenoptera acutorostrata scammoni	103010181	Model	Mammal
Physeter catodon	102975759	Model	Mammal
Orcinus orca	101284384	Model	Mammal
Tursiops truncatus	101328434	Provisional	Mammal
Alligator mississippiensis	NA		Reptile
Gavialis gangeticus	NA		Reptile
Chelonia mydas	102932918	Model	Reptile
Xenopus tropicalis	NA		Amphibian
Nanorana parkeri	NA		Amphibian
Gallus gallus	100861585	Proivisional	Bird
Columba Livia	NA		Bird
Coturnix japonica	NA		Bird
Numida meleagris	NA		Bird
Danio rerio	NA		Bony fish
Cyprinodon variegatus	NA		Bony fish
Poecilia formosa	NA		Bony fish
Oncorhynchus mykiss	NA		Bony fish
Callorhinchus Milii	NA		Cartilaginous fish
Salmo salar	NA		Bony fish
Cyprinus carpio	NA		Bony fish
Labrus bergylta	NA		Bony fish
Scyliorhinus canicula	NA		Cartilaginous fish
**IL36RN**			
Homo sapiens	26525	Reviewed	Mammal
Macaca fascicularis	102124609	Model	Mammal
Bos taurus	518514	Provisional	Mammal
Rattus norvegicus	311783	Provisional	Mammal
Canis lupus familiaris	611869	Model	Mammal
Equus caballus	100065154	Model	Mammal
Mus musculus	54450	Validated	Mammal
Sus scrofa	NA		Mammal
Ovis Aries	101111354	Model	Mammal
Felis catus	NA		Mammal
Ornithorhynchus anatinus	NA		Mammal
Sarcophilus harrisii	100923393	Model	Mammal
Monodelphis domestica	100026133	Model	Mammal
Balaenoptera acutorostrata scammoni	103020502	Model	Mammal
Physeter catodon	102975470	Model	Mammal
Orcinus orca	101284641	Model	Mammal
Tursiops truncatus	NA		Mammal
Alligator mississippiensis	106738033	Model	Reptile
Gavialis gangeticus	NA		Reptile
Chelonia mydas	NA		Reptile
Xenopus tropicalis	NA		Amphibian
Nanorana parkeri	NA		Amphibian
Gallus gallus	100861586	Model	Bird
Columba Livia	NA		Bird
Coturnix japonica	NA		Bird

Numida meleagris	NA		Bird
Danio rerio	NA		Bony fish
Cyprinodon variegatus	NA		Bony fish
Poecilia formosa	NA		Bony fish
Oncorhynchus mykiss	NA		Bony fish
Callorhinchus Milii	NA		Cartilaginous fish
Salmo salar	NA		Bony fish
Cyprinus carpio	NA		Bony fish
Labrus bergylta	NA		Bony fish
Scyliorhinus canicula	NA		Cartilaginous fish

**IL36RN**			
Homo sapiens	26525	Reviewed	Mammal
Macaca fascicularis	102124609	Model	Mammal
Bos taurus	518514	Provisional	Mammal
Rattus norvegicus	311783	Provisional	Mammal
Canis lupus familiaris	611869	Model	Mammal
Equus caballus	100065154	Model	Mammal
Mus musculus	54450	Validated	Mammal
Sus scrofa	NA		Mammal
Ovis Aries	101111354	Model	Mammal
Felis catus	NA		Mammal
Ornithorhynchus anatinus	NA		Mammal
Sarcophilus harrisii	100923393	Model	Mammal
Monodelphis domestica	100026133	Model	Mammal
Balaenoptera acutorostrata scammoni	103020502	Model	Mammal
Physeter catodon	102975470	Model	Mammal
Orcinus orca	101284641	Model	Mammal
Tursiops truncatus	NA		Mammal
Alligator mississippiensis	106738033	Model	Reptile
Gavialis gangeticus	NA		Reptile
Chelonia mydas	NA		Reptile
Xenopus tropicalis	NA		Amphibian
Nanorana parkeri	NA		Amphibian
Gallus gallus	100861586	Model	Bird
Columba Livia	NA		Bird
Coturnix japonica	NA		Bird
Numida meleagris	NA		Bird
Danio rerio	NA		Bony fish
Cyprinodon variegatus	NA		Bony fish
Poecilia formosa	NA		Bony fish
Oncorhynchus mykiss	NA		Bony fish
Callorhinchus Milii	NA		Cartilaginous fish
Salmo salar	NA		Bony fish
Cyprinus carpio	NA		Bony fish
Labrus bergylta	NA		Bony fish
Scyliorhinus canicula	NA		Cartilaginous fish

**FGF1**			
Homo sapiens	2246	Reviewed	Mammal

Macaca fascicularis	102134593	Model	Mammal
Bos taurus	281160	Model	Mammal
Rattus norvegicus	25317	Provisional	Mammal
Canis lupus familiaris	607724	Model	Mammal
Equus caballus	100033960	Model	Mammal
Mus musculus	14164	Validated	Mammal
Sus scrofa	397497	Model	Mammal
Ovis Aries	101109353	Model	Mammal
Felis catus	101082206	Model	Mammal
Ornithorhynchus anatinus	100078822	Model	Mammal
Sarcophilus harrisii	100929695	Model	Mammal
Monodelphis domestica	100023197	Model	Mammal
Balaenoptera acutorostrata scammoni	103018997	Model	Mammal
Physeter catodon	NA	Model	Mammal
Orcinus orca	101277714	Model	Mammal
Tursiops truncatus	101327652	Model	Mammal
Alligator mississippiensis	102570160	Model	Reptile
Gavialis gangeticus	109289995	Model	Reptile
Chelonia mydas	102931997	Model	Reptile
Xenopus tropicalis	100217324	Provisional	Amphibian
Nanorana parkeri	108794559	Model	Amphibian
Gallus gallus	396094	Model	Bird
Columba Livia	102091153	Model	Bird
Coturnix japonica	107320055	Model	Bird
Numida meleagris	110405391	Model	Bird
Danio rerio	393733	Provisional	Bony fish
Cyprinodon variegatus	NA	Model	Bony fish
Poecilia formosa	103475366	Model	Bony fish
Oncorhynchus mykiss	103184127	Model	Bony fish
Callorhinchus Milii	103184127	Model	Cartilaginous fish
Salmo salar	100196882	Model	Bony fish
Cyprinus carpio	109109517	Model	Bony fish
Labrus bergylta	109995648	Model	Bony fish
Scyliorhinus canicula	NA	Model	Cartilaginous fish

**FGF2**			
Homo sapiens	2247	Reviewed	Mammal
Macaca fascicularis	NA		Mammal
Bos taurus	281161	Validated	Mammal
Rattus norvegicus	54250	Validated	Mammal
Canis lupus familiaris	NA	Model	Mammal
Equus caballus	100033955	Model	Mammal
Mus musculus	14173	Validated	Mammal
Sus scrofa	397643	Model	Mammal
Ovis Aries	443306	Provisional	Mammal
Felis catus	100135772	Model	Mammal
Ornithorhynchus anatinus	NA		Mammal
Sarcophilus harrisii	100934542	Model	Mammal
Monodelphis domestica	619384	Provisional	Mammal

Balaenoptera acutorostrata scammoni	103015722	Model	Mammal
Physeter catodon	102989017	Model	Mammal
Orcinus orca	101280803	Model	Mammal
Tursiops truncatus	101338598	Model	Mammal
Alligator mississippiensis	102567370	Model	Reptile
Gavialis gangeticus	109301132	Model	Reptile
Chelonia mydas	102931444	Model	Reptile
Xenopus tropicalis	550087	Model	Amphibian
Nanorana parkeri	108790780	Model	Amphibian
Gallus gallus	396413	Model	Bird
Columba Livia	110364865	Model	Bird
Coturnix japonica	10313543	Model	Bird
Numida meleagris	NA	Model	Bird
Danio rerio	404231	Provisional	Bony fish
Cyprinodon variegatus	107099215	Model	Bony fish
Poecilia formosa	103137295	Model	Bony fish
Oncorhynchus mykiss	100136280	Provisional	Bony fish
Callorhinchus Milii	103188323	Model	Cartilaginous fish
Salmo salar	106611625	Model	Bony fish
Cyprinus carpio	NA	Model	Bony fish
Labrus bergylta	109999743	Model	Bony fish
Scyliorhinus canicula		Model	Cartilaginous fish

**FGF4**			
Homo sapiens	2249	Reviewed	Mammal
Macaca fascicularis	102134588	Model	Mammal
Bos taurus	618474	Provisional	Mammal
Rattus norvegicus	116499	Model	Mammal
Canis lupus familiaris	483680	Model	Mammal
Equus caballus	NA	Model	Mammal
Mus musculus	14175	Validated	Mammal
Sus scrofa	100518595	Model	Mammal
Ovis Aries musimon	101116004	Model	Mammal
Felis catus	NA		Mammal
Ornithorhynchus anatinus	100075228	Model	Mammal
Sarcophilus harrisii	100921812	Model	Mammal
Monodelphis domestica	100021631	Model	Mammal
Balaenoptera acutorostrata scammoni	NA		Mammal
Physeter catodon	NA		Mammal
Orcinus orca	101273794	Model	Mammal
Tursiops truncatus	101331363	Model	Mammal
Alligator mississippiensis	102563000	Model	Reptile
Gavialis gangeticus	109298992	Model	Reptile
Chelonia mydas	NA		Reptile
Xenopus tropicalis	100217325	Provisional	Amphibian
Nanorana parkeri	108785455	Model	Amphibian
Gallus gallus	428857	Validated	Bird
Columba Livia	102095791	Model	Bird
Coturnix japonica	107314467	Model	Bird

Numida meleagris	110401571	Model	Bird
Danio rerio	570435	Validated	Bony fish
Cyprinodon variegatus	107096214	Model	Bony fish
Poecilia formosa	103150952	Model	Bony fish
Oncorhynchus mykiss	NA		Bony fish
Callorhinchus Milii	103175016	Model	Cartilaginous fish
Salmo salar	NA		Bony fish
Cyprinus carpio	758818084	Model	Bony fish
Labrus bergylta	NA		Bony fish
Scyliorhinus canicula	NA		Cartilaginous fish

**FGF7**			
Homo sapiens	2252	Reviewed	Mammal
Macaca fascicularis	102131922	Model	Mammal
Bos taurus	616885	Provisional	Mammal
Rattus norvegicus	29348	Model	Mammal
Canis lupus familiaris	403915	Model	Mammal
Equus caballus	100033961	Model	Mammal
Mus musculus	14178	Validated	Mammal
Sus scrofa	397281	Model	Mammal
Ovis Aries	443095	Model	Mammal
Felis catus	101097982	Model	Mammal
Ornithorhynchus anatinus	NA		Mammal
Sarcophilus harrisii	100925383	Model	Mammal
Monodelphis domestica	100025923	Model	Mammal
Balaenoptera acutorostrata scammoni	103020278	Model	Mammal
Physeter catodon	102994154	Model	Mammal
Orcinus orca	101288260	Model	Mammal
Tursiops truncatus	101322398	Model	Mammal
Alligator mississippiensis	109280173	Model	Reptile
Gavialis gangeticus	109288585	Model	Reptile
Chelonia mydas	NA		Reptile
Xenopus tropicalis	734697	Provisional	Amphibian
Nanorana parkeri	108792194	Model	Amphibian
Gallus gallus	415439	Model	Bird
Columba Livia	102087779	Model	Bird
Coturnix japonica	107318735	Model	Bird
Numida meleagris	110404057	Model	Bird
Danio rerio	493181	Model	Bony fish
Cyprinodon variegatus	107085362	Model	Bony fish
Poecilia formosa	103462084	Model	Bony fish
Oncorhynchus mykiss	NA		Bony fish
Callorhinchus Milii	103186971	Provisional	Cartilaginous fish
Salmo salar	NA		Bony fish
Cyprinus carpio	NA		Bony fish
Labrus bergylta	109989468	Model	Bony fish
Scyliorhinus canicula	NA		Cartilaginous fish

**FGF9**			
Homo sapiens	2254	Reviewed	Mammal

Macaca fascicularis	102140725	Model	Mammal
Bos taurus	613731	Provisional	Mammal
Rattus norvegicus	25444	Provisional	Mammal
Canis lupus familiaris	477340	Model	Mammal
Equus caballus	100050353	Model	Mammal
Mus musculus	14180	Validated	Mammal
Sus scrofa	396717	Provisional	Mammal
Ovis Aries	101116844	Model	Mammal
Felis catus	101084335	Model	Mammal
Ornithorhynchus anatinus	100089319	Model	Mammal
Sarcophilus harrisii	100920456	Model	Mammal
Monodelphis domestica	NA		Mammal
Balaenoptera acutorostrata scammoni	103004048	Model	Mammal
Physeter catodon	102990012	Model	Mammal
Orcinus orca	101284280	Model	Mammal
Tursiops truncatus	101322077	Model	Mammal
Alligator mississippiensis	102577004	Model	Reptile
Gavialis gangeticus	109303266	Model	Reptile
Chelonia mydas	102933711	Model	Reptile
Xenopus tropicalis	378562	Provisional	Amphibian
Nanorana parkeri	108793985	Model	Amphibian
Gallus gallus	378917	Provisional	Bird
Columba Livia	102098285	Model	Bird
Coturnix japonica	107308312	Model	Bird
Numida meleagris	110389317	Model	Bird
Danio rerio	NA		Bony fish
Cyprinodon variegatus	NA		Bony fish
Poecilia formosa	NA		Bony fish
Oncorhynchus mykiss	NA		Bony fish
Callorhinchus Milii	103183275	Model	Cartilaginous fish
Salmo salar	NA	Model	Bony fish
Cyprinus carpio	NA	Model	Bony fish
Labrus bergylta	NA	Model	Bony fish
Scyliorhinus canicula	NA	Model	Cartilaginous fish

**FGF10**			
Homo sapiens	602115	Reviewed	Mammal
Macaca fascicularis	102143550	Provisional	Mammal
Bos taurus	326285	Provisional	Mammal
Rattus norvegicus	25443	Provisional	Mammal
Canis lupus familiaris	612454	Model	Mammal
Equus caballus	100052655	Model	Mammal
Mus musculus	14165	Validated	Mammal
Sus scrofa	100525086	Model	Mammal
Ovis Aries	443074	Provisional	Mammal
Felis catus	101090489	Model	Mammal
Ornithorhynchus anatinus	NA		Mammal
Sarcophilus harrisii	NA		Mammal
Monodelphis domestica	100012692	Model	Mammal

Balaenoptera acutorostrata scammoni	NA		Mammal
Physeter catodon	NA		Mammal
Orcinus orca	101281389	Model	Mammal
Tursiops truncatus	NA		Mammal
Alligator mississippiensis	102568685	Model	Reptile
Gavialis gangeticus	109297843	Model	Reptile
Chelonia mydas	102934890	Model	Reptile
Xenopus tropicalis	548923	Model	Amphibian
Nanorana parkeri	108796567	Model	Amphibian
Gallus gallus	395432	Provisional	Bird
Columba Livia	110357664	Model	Bird
Coturnix japonica	107305970	Model	Bird
Numida meleagris	NA		Bird
Danio rerio	359830	Provisional	Bony fish
Cyprinodon variegatus	107089663	Model	Bony fish
Poecilia formosa	103469999	Model	Bony fish
Oncorhynchus mykiss	NA		Bony fish
Callorhinchus Milii	103177849	Model	Cartilaginous fish
Salmo salar	106580601	Model	Bony fish
Cyprinus carpio	NA		Bony fish
Labrus bergylta	NA		Bony fish
Scyliorhinus canicula	NA	Model	Cartilaginous fish

**FGF19**			
Homo sapiens	603891	Reviewed	Mammal
Macaca fascicularis	102134957	Model	Mammal
Bos taurus	521475	Provisional	Mammal
Rattus norvegicus	620166	Provisional	Mammal
Canis lupus familiaris	483681	Model	Mammal
Equus caballus	NA	Model	Mammal
Mus musculus	NA	Validated	Mammal
Sus scrofa	100518950	Model	Mammal
Ovis Aries	101115744	Model	Mammal
Felis catus	101087099	Model	Mammal
Ornithorhynchus anatinus	100075189	Model	Mammal
Sarcophilus harrisii	NA		Mammal
Monodelphis domestica	100021575	Model	Mammal
Balaenoptera acutorostrata scammoni	103012083	Model	Mammal
Physeter catodon	102974372	Model	Mammal
Orcinus orca	101272704	Model	Mammal
Tursiops truncatus	101335071	Model	Mammal
Alligator mississippiensis	102563235	Model	Reptile
Gavialis gangeticus	109298993	Model	Reptile
Chelonia mydas	102948383	Model	Reptile
Xenopus tropicalis	100217329	Model	Amphibian
Nanorana parkeri	108785439	Model	Amphibian
Gallus gallus	395394	Validated	Bird
Columba Livia	102095975	Model	Bird
Coturnix japonica	107314429	Model	Bird

Numida meleagris	110400908	Model	Bird
Danio rerio	368245	Model	Bony fish
Cyprinodon variegatus	NA		Bony fish
Poecilia formosa	103462294	Model	Bony fish
Oncorhynchus mykiss	110526225	Model	Bony fish
Callorhinchus Milii	103175015	Model	Cartilaginous fish
Salmo salar	NA		Bony fish
Cyprinus carpio	109049194	Model	Bony fish
Labrus bergylta	109982309	Model	Bony fish
Scyliorhinus canicula	NA		Cartilaginous fish

**HAX-1**			
Homo sapiens	10456	Reviewed	Mammal
Macaca fascicularis	102119274	Model	Mammal
Bos taurus	506895	Model	Mammal
Rattus norvegicus	NA		Mammal
Canis lupus familiaris	480134	Model	Mammal
Equus caballus	100056899	Model	Mammal
Mus musculus	23897	Validated	Mammal
Sus scrofa	100739544	Model	Mammal
Ovis Aries	101111760	Model	Mammal
Felis catus	101097787	Model	Mammal
Ornithorhynchus anatinus	100089567	Model	Mammal
Sarcophilus harrisii	100918638	Model	Mammal
Monodelphis domestica	100015562	Model	Mammal
Balaenoptera acutorostrata scammoni	102997412	Model	Mammal
Physeter catodon	102993879	Model	Mammal
Orcinus orca	101280540	Model	Mammal
Tursiops truncatus	101339719	Model	Mammal
Alligator mississippiensis	102570010	Model	Reptile
Gavialis gangeticus	109304128	Model	Reptile
Chelonia mydas	102948001	Model	Reptile
Xenopus tropicalis	100485912	Model	Amphibian
Nanorana parkeri	108799655	Model	Amphibian
Gallus gallus	107055138	Model	Bird
Columba Livia	NA		Bird
Coturnix japonica	107324498	Model	Bird
Numida meleagris	110387961	Model	Bird
Danio rerio	436609	Provisional	Bony fish
Cyprinodon variegatus	107101122	Model	Bony fish
Poecilia formosa	103130962	Model	Bony fish
Oncorhynchus mykiss	110495990	Model	Bony fish
Callorhinchus Milii	24402279	Model	Cartilaginous fish
Salmo salar	100194877	Provisional	Bony fish
Cyprinus carpio	NA	Model	Bony fish
Labrus bergylta	10995026	Model	Bony fish
Scyliorhinus canicula	NA	Model	Cartilaginous fish
Pseudomyrmex gracilis	109852091	Model	Arthropod
Cephus cinctus	107271917	Model	Arthropod
Fopius arisanus	105273834	Model	Arthropod

Crassostrea gigas	105340833	Model	Mollusca

Taxa	Gene ID	RefSeq status	Clade
**IL1R1**			
Homo sapien	3554	Reviewed	Mammal
Macaca fascicularis	102146317	Model	Mammal
Bos taurus	515640	Validated	Mammal
Rattus norvegicus	25663	Provisional	Mammal
Canis lupus familiaris	481328	Model	Mammal
Equus caballus	100009699	Validated	Mammal
Mus musculus	16177	Validated	Mammal
Sus scrofa	100626904	Model	Mammal
Ovis Aries	101103054	Model	Mammal
Felis catus	101080705	Model	Mammal
Ornithorhynchus anatinus	100084073	Model	Mammal
Sarcophilus harrisii	100913514	Model	Mammal
Monodelphis domestica	100017751	Model	Mammal
Balaenoptera acutorostrata scammoni	102998234	Model	Mammal
Physeter catodon	102973768	Model	Mammal
Orcinus orca	101277609	Model	Mammal
Tursiops truncatus	101329065	Model	Mammal
Alligator mississippiensis	NA		Reptile
Gavialis gangeticus	NA		Reptile
Chelonia mydas	NA		Reptile
Xenopus tropicalis	100491546	Model	Amphibian
Nanorana parkeri	NA		Amphibian
Gallus gallus	396481	Provisional	Bird
Columba ivia	102097520	Model	Bird
Coturnix japonica	NA		Bird
Numida meleagris	NA		Bird
Danio rerio	NA		Bony fish
Cyprinus carpio	NA		Bony fish
Mesocricetus auratus	101823176	Model	Bony Fish
Oncorhynchus mykiss	NA		Bony fish
Callorhinchus Milii	NA		Cartilaginous fish
Salmo salar	100136530	Model	Bony fish
Cyprinus carpio	NA		Bony Fish
Labrus bergylta	NA		Bony Fish
**IL1R2**			
Homo sapiens	7850	Reviewed	Mammal
Macaca fascicularis	102143926	Provisional	Mammal
Bos taurus	515700	Provisional	Mammal
Rattus norvegicus	117022	Provisional	Mammal
Canis lupus familiaris	481330	Model	Mammal
Equus caballus	100033831	Provisional	Mammal
Mus musculus	16178	Provisional	Mammal
Sus scrofa	100628112	Model	Mammal
Tursiops truncatus	101337838	Model	Mammal
Ovis Aries	101103308	Model	Mammal

Felis catus	101080961	Model	Mammal
Sarcophilus harrisii	100913252	Model	Mammal
Monodelphis domestica	100017640	Model	Mammal
Balaenoptera acutorostrata scammoni	102999629	Model	Mammal
Physeter catodon	NA	Model	Mammal
Orcinus orca	101277609	Model	Mammal
Tursiops truncatus	101337838	Model	Mammal
Alligator mississippiensis	1011604302	Model	Reptile
Gavialis gangeticus	NA		Reptile
Chelonia mydas	102937756	Model	Reptile
Xenopus tropicalis	548430	Model	Amphibian
Nanorana parkeri	108785107	Model	Amphibian
Gallus gallus	418715	Model	Bird
Columba Livia	NA		Bird
Coturnix japonica	107306854	Model	Bird
Numida meleagris	110406769	Model	Bird
Danio rerio	NA		Bony fish
Cyprinodon variegatus	NA		Bony fish
Poecilia formosa	NA		Bony fish
Oncorhynchus mykiss	NA		Bony fish
Callorhinchus Milii	NA		Cartilaginous fish
Salmo salar	100270808	Provisional	Bony fish
Cyprinus carpio	NA		Bony fish
Labrus bergylta	109974022	Model	Bony fish

**IL1R3/IL-1RAcP**			
Homo sapiens	3556	REVIEWED	Mammal
Macaca fascicularis	102133222	Model	Mammal
Bos taurus	539334	Model	Mammal
Rattus norvegicus	2893	PROVISIONAL	Mammal
Canis lupus familiaris	488126	Model	Mammal
Equus caballus	100068726	Model	Mammal
Mus musculus	16180	REVIEWED	Mammal
Sus scrofa	100628318	Model	Mammal
Ovis Aries	101108354	Model	Mammal
Felis catus	101094125	Model	Mammal
Ornithorhynchus anatinus	NA		Mammal
Sarcophilus harrisii	100933140	Model	Mammal
Monodelphis domestica	100016835	Model	Mammal
Balaenoptera acutorostrata scammoni	103002906	Model	Mammal
Physeter catodon	102994287	Model	Mammal
Orcinus orca	101285541	Model	Mammal
Tursiops truncatus	101335680	Model	Mammal
Alligator mississippiensis	102567774	Model	Reptile
Gavialis gangeticus	NA		Reptile
Chelonia mydas	102940652	Model	Reptile
Xenopus tropicalis	100216065	Model	Amphibian
Nanorana parkeri	108804591	Model	Amphibian
Gallus gallus	424908	Model	Bird
Columba Livia	102092020	Model	Bird

Coturnix japonica	107318248	Model	Bird
Numida meleagris	NA		Bird
Danio rerio	100331351	Model	Bony fish
Cyprinodon variegatus	107086872	Model	Bony fish
Poecilia formosa	103150889	Model	Bony fish
Oncorhynchus mykiss	NA		Bony fish
Callorhinchus Milii	NA		Cartilaginous fish
Salmo salar	NA		Bony fish
Cyprinus carpio	NA		Bony fish
Labrus bergylta	NA		Bony fish

**IL1R4/IL-1RL1/ST2**			
Homo sapiens	9173	REVIEWED	Mammal
Macaca fascicularis	102115135	Model	Mammal
Bos taurus	520709	Model	Mammal
Rattus norvegicus	25556	Model	Mammal
Canis lupus familiaris	611442	Model	Mammal
Equus caballus	100058357	Model	Mammal
Mus musculus	17082	Validated	Mammal
Sus scrofa	NA	Model	Mammal
Ovis Aries	101109529	Model	Mammal
Felis catus	101080448	Model	Mammal
Ornithorhynchus anatinus	NA	Model	Mammal
Sarcophilus harrisii	100914043	Model	Mammal
Monodelphis domestica	100017856	Model	Mammal
Balaenoptera acutorostrata scammoni	103013226	Model	Mammal
Physeter catodon	102974232	Model	Mammal
Orcinus orca	102974232	Model	Mammal
Tursiops truncatus	101316920	Model	Mammal
Alligator mississippiensis	102560559	Model	Reptile
Gavialis gangeticus	NA		Reptile
Chelonia mydas	102944780	Model	Reptile
Xenopus tropicalis	xxxx	Model	Amphibian
Nanorana parkeri	xxxx	Model	Amphibian
Gallus gallus	374136	Model	Bird
Columba Livia	102097335	Model	Bird
Coturnix japonica	107306743	Model	Bird
Numida meleagris	NA		Bird
Danio rerio	407649	Model	Bony fish
Cyprinodon variegatus	NA	Model	Bony fish
Poecilia formosa	NA	Model	Bony fish
Oncorhynchus mykiss	526253038	PROVISIONAL	Bony fish
Callorhinchus Milii	NA		Cartilaginous fish
Salmo salar	NA		
Cyprinus carpio	NA		
Labrus bergylta	NA		

**IL1R5/IL-118R**			
Homo sapiens	4504655	REVIEWED	Mammal
Macaca fascicularis	544479591	Model	Mammal

Bos taurus	528967972	Model	Mammal
Rattus norvegicus	301365	Model	Mammal
Canis lupus familiaris	301365	Model	Mammal
Equus caballus	100058269	Model	Mammal
Mus musculus	16182	Validated	Mammal
Sus scrofa	397179	Model	Mammal
Ovis Aries	101102552	Model	Mammal
Felis catus	493938	VALIDATED	Mammal
Ornithorhynchus anatinus	NA		Mammal
Sarcophilus harrisii	100914298	Model	Mammal
Monodelphis domestica	100017899	Model	Mammal
Balaenoptera acutorostrata scammoni	103012740	Model	Mammal
Physeter catodon	102973670	Model	Mammal
Orcinus orca	101275947	Model	Mammal
Tursiops truncatus	101331669	Model	Mammal
Alligator mississippiensis	102560330	Model	Reptile
Gavialis gangeticus	NA		
Chelonia mydas	102944555	Model	Reptile
Xenopus tropicalis	NA		Amphibian
Nanorana parkeri	NA		Amphibian
Gallus gallus	418717	Model	Bird
Columba Livia	102085680	Model	Bird
Coturnix japonica	107306721	Model	Bird
Numida meleagris	NA		Bird
Danio rerio	NA		Bony fish
Cyprinodon variegatus	NA		Bony fish
Poecilia formosa	NA		Bony fish
Oncorhynchus mykiss	NA		Bony fish
Callorhinchus Milii	103172473	Model	Cartilaginous fish
NA			
NA			
NA			

**IL1R6/IL-1RL2**			
Homo sapiens	8808	REVIEWED	Mammal
Macaca fascicularis	102145305	Model	Mammal
Bos taurus	522114	Model	Mammal
Rattus norvegicus	621782	Model	Mammal
Canis lupus familiaris	611453	Model	Mammal
Equus asinus	106839401	Model	Mammal
Mus musculus	107527	Validated	Mammal
Sus scrofa	106509756	Model	Mammal
Ovis Aries	101102800	Model	Mammal
Felis catus	101085113	Model	Mammal
Ornithorhynchus anatinus	NA		Mammal
Sarcophilus harrisii	100913777	Model	Mammal
Monodelphis domestica	100017791	Model	Mammal
Balaenoptera acutorostrata scammoni	103013514	Model	Mammal
Physeter catodon	102974910	Model	Mammal
Orcinus orca	101276455	Model	Mammal

Tursiops truncatus	101333629	Model	Mammal
Alligator mississippiensis	NA		Reptile
Gavialis gangeticus	109303181	Model	Reptile
Chelonia mydas	102945219	Model	Reptile
Xenopus tropicalis	NA		Amphibian
Nanorana parkeri	NA		Amphibian
Gallus gallus	418716	Model	Bird
Columba Livia	NA		Bird
Coturnix japonica	NA		Bird
Numida meleagris	NA		Bird
Danio rerio	NA		Bony fish
Cyprinodon variegatus	NA		Bony fish
Poecilia formosa	NA		Bony fish
Oncorhynchus mykiss	NA		Bony fish
Callorhinchus Milii	NA		Cartilaginous fish
Salmo salar	NA		Bony fish
Cyprinus carpio	NA		Bony fish
Labrus bergylta	NA		Bony fish

**IL1R7/IL18RAP**			
Homo sapiens	8807	REVIEWED	Mammal
Macaca fascicularis	712046	Model	Mammal
Bos taurus	493716	Model	Mammal
Rattus norvegicus	727867	Model	Mammal
Canis lupus familiaris	481327	Model	Mammal
Equus caballus	100050212	Model	Mammal
Mus musculus	16174	REVIEWED	Mammal
Sus scrofa	NA	Model	Mammal
Ovis Aries	101109262	Model	Mammal
Felis catus	101084868	Model	Mammal
Ornithorhynchus anatinus	NA	Model	Mammal
Sarcophilus harrisii	100929823	Model	Mammal
Monodelphis domestica	100017931	Model	Mammal
Balaenoptera acutorostrata scammoni	103012459	Model	Mammal
Physeter catodon	NA	Model	Mammal
Orcinus orca	101277121	Model	Mammal
Tursiops truncatus	101331381	Model	Mammal
Alligator mississippiensis	102560095	Model	Reptile
Gavialis gangeticus	109303410	Model	Reptile
Chelonia mydas	NA	Model	Reptile
Xenopus tropicalis	100491212	Model	Amphibian
Nanorana parkeri	108785111	Model	Amphibian
Gallus gallus	101750396	Model	Bird
Columba Livia	102085495	Model	Bird
Coturnix japonica	107307916	Model	Bird
Numida meleagris	NA		Bird
Danio rerio	NA		Bony fish
Cyprinodon variegatus	NA		Bony fish
Poecilia formosa	NA		Bony fish
Oncorhynchus mykiss	NA		Bony fish

Callorhinchus Milii	103172467	Model	Cartilaginous fish
Salmo salar	NA		Bony fish
Cyprinus carpio	NA		Bony fish
Labrus bergylta	NA		Bony fish

IL1R8/SIGRR			
Homo sapiens	59307	Validated	Mammal
Macaca fascicularis	101864854	Model	Mammal
Bos taurus	531801	Model	Mammal
Rattus norvegicus	309106	Model	Mammal
Canis lupus familiaris	100686933	Model	Mammal
Equus caballus	100054544	Model	Mammal
Mus musculus	24058	Validated	Mammal
Sus scrofa	100626800	Provisional	Mammal
Ovis Aries	101104191	Model	Mammal
Felis catus	101100416	Model	Mammal
Ornithorhynchus anatinus	NA	Model	Mammal
Sarcophilus harrisii	100921726	Model	Mammal
Monodelphis domestica	NA	Model	Mammal
Balaenoptera acutorostrata scammoni	103008982	Model	Mammal
Physeter catodon	102989861	Model	Mammal
Orcinus orca	101286986	Model	Mammal
Tursiops truncatus	NA	Model	Mammal
Alligator mississippiensis	102569037	Model	Reptile
Gavialis gangeticus	109299250	Model	Reptile
Chelonia mydas	102929383	Model	Reptile
Xenopus tropicalis	100486638	Model	Amphibian
Nanorana parkeri	108791997	Model	Amphibian
Gallus gallus	422995	Model	Bird
Columba Livia	102084113	Model	Bird
Coturnix japonica	107314267	Model	Bird
Numida meleagris	110401651	Model	Bird
Danio rerio	563606	Provisional	Bony fish
Cyprinodon variegatus	NA	Model	Bony fish
Poecilia formosa	103156514	Model	Bony fish
Oncorhynchus mykiss	110501108	Model	Bony fish
Callorhinchus Milii	NA	Model	Cartilaginous fish
Salmo salar	100380553	Model	Bony fish
Cyprinus carpio	109070341	Model	Bony fish
Labrus bergylta	110003576	Model	Bony fish

IL1R9/IL1RAPL1			
Homo sapiens	11141	Reviewed	Mammal
Macaca fascicularis	102133143	Model	Mammal
Bos taurus	541298	Model	Mammal
Rattus norvegicus	317553	Provisional	Mammal
Canis lupus familiaris	491800	Model	Mammal
Equus caballus	100052404	Model	Mammal
Mus musculus	331461	Validated	Mammal
Sus scrofa	100621199	Model	Mammal

Ovis Aries	101117137	Model	Mammal
Felis catus	101082883	Model	Mammal
Ornithorhynchus anatinus	100074745	Model	Mammal
Sarcophilus harrisii	100923060	Model	Mammal
Monodelphis domestica	100009941	Model	Mammal
Balaenoptera acutorostrata scammoni	103004696	Model	Mammal
Physeter catodon	NA		Mammal
Orcinus orca	101270975	Model	Mammal
Tursiops truncatus	101319470	Model	Mammal
Alligator mississippiensis	106737134	Model	Reptile
Gavialis gangeticus	109302796	Model	Reptile
Chelonia mydas	102932666	Model	Reptile
Xenopus tropicalis	100486886	Model	Amphibian
Nanorana parkeri	108792678	Model	Amphibian
Gallus gallus	427993	Model	Bird
Columba Livia	102099046	Model	Bird
Coturnix japonica	107324825	Model	Bird
Numida meleagris	NA		Bird
Danio rerio	568868	Model	Bony fish
Cyprinodon variegatus	107090101	Model	Bony fish
Poecilia formosa	103140382	Model	Bony fish
Oncorhynchus mykiss	110495483	Model	Bony fish
Callorhinchus Milii	NA		Cartilaginous fish
Salmo salar	NA		Bony fish
Cyprinus carpio	NA		Bony fish
Labrus bergylta	NA		Bony fish
**IL1R10/IL1RAPL2**			
Homo sapiens	300277	Reviewed	Mammal
Macaca fascicularis	102139365	Model	Mammal
Bos taurus	523444	Model	Mammal
Rattus norvegicus	1561761	Model	Mammal
Canis lupus familiaris	NA		Mammal
Equus caballus	NA		Mammal
Mus musculus	60367	Validated	Mammal
Sus scrofa	100157100	Model	Mammal
Ovis Aries	101101957	Model	Mammal
Felis catus	101100037	Model	Mammal
Ornithorhynchus anatinus	100082564	Model	Mammal
Sarcophilus harrisii	100929692	Model	Mammal
Monodelphis domestica	100009903	Model	Mammal
Balaenoptera acutorostrata scammoni	103020523	Model	Mammal
Physeter catodon	102973597	Model	Mammal
Orcinus orca	101271660	Model	Mammal
Tursiops truncatus	101335027	Model	Mammal
Alligator mississippiensis	102568377	Model	Reptile
Gavialis gangeticus	109300794	Model	Reptile
Chelonia mydas	102934844	Model	Reptile

Xenopus tropicalis	779820	Model	Amphibian
Nanorana parkeri	108788660	Model	Amphibian
Gallus gallus	422379	Model	Bird
Columba Livia	102088910	Model	Bird
Coturnix japonica	107312960	Model	Bird
Numida meleagris	110403365	Model	Bird
Danio rerio	100149452	Provisional	Bony fish
Cyprinodon variegatus	NA	Model	Bony fish
Poecilia formosa	NA	Model	Bony fish
Oncorhynchus mykiss	NA	Model	Bony fish
Callorhinchus Milii	103179448	Model	Cartilaginous fish
Salmo salar	NA	Model	Bony fish
Cyprinus carpio	NA	Model	Bony fish
Labrus bergylta	NA	Model	Bony fish

**IL18BP**			
Homo sapiens	10068	Model	Mammal
Macaca fascicularis	107126419	Model	Mammal
Bos taurus	617470	Model	Mammal
Rattus norvegicus	620042	Model	Mammal
Canis lupus familiaris	476818	Model	Mammal
Equus caballus	100066161	Model	Mammal
Mus musculus	16068	Model	Mammal
Sus scrofa	100525765	Model	Mammal
Ovis Aries	101118983	Model	Mammal
Felis catus	101096599	Model	Mammal
Ornithorhynchus anatinus	NA		Mammal
Sarcophilus harrisii	NA		Mammal
Monodelphis domestica	103102783	Model	Mammal
Balaenoptera acutorostrata scammoni	103019816	Model	Mammal
Physeter catodon	102995238	Model	Mammal
Orcinus orca	101272128	Model	Mammal
Tursiops truncatus	101332181	Model	Mammal
Alligator mississippiensis	NA		Reptile
Gavialis gangeticus	NA		Reptile
Chelonia mydas	102945076	Model	Reptile
Xenopus tropicalis	NA		Amphibian
Nanorana parkeri	NA		Amphibian
Gallus gallus	107052452	Model	Bird
Columba Livia	NA		Bird
Coturnix japonica	107308522	Model	Bird
Numida meleagris	NA		Bird
Danio rerio	NA		Bony fish
Cyprinodon variegatus	NA		Bony fish
Poecilia formosa	NA		Bony fish
Oncorhynchus mykiss	NA		Bony fish
Callorhinchus Milii	NA		Cartilaginous fish
Salmo salar	106567866	Model	Bony fish
Cyprinus carpio	NA		Bony fish
Labrus bergylta	NA		Bony fish
