# Supplementary material for: Redefining the ancestral origins of the interleukin-1 superfamily
Source: Nat Commun. 2018 Mar 20;9:1156. doi: 10.1038/s41467-018-03362-1 (PMC5861070; doi:10.1038/s41467-018-03362-1)
Supplement: Supplementary file 11 — Description of Additional Supplementary Files(PDF 188 kb) [file 41467_2018_3362_MOESM11_ESM.pdf]

Description of Additional Supplementary Files

File Name: Supplementary Data 1
Description: Molecular phylogenetic analysis of the IL-1 family of receptors. The percentage of trees in which the associated group clustered together is shown next to the branches from 1000 bootstrap replications. Branches which occur in less than 50% of trees were collapsed. The tree is drawn to scale, with branch lengths measured in the number of amino acid replacements per site. The analysis involved 231 amino acid sequences with an alignment length of 64 positions in the final dataset. All positions containing gaps and missing data were eliminated.

File Name: Supplementary Data 2
Description: Molecular phylogenetic analysis by of the IL-1 family of cytokines. The percentage of trees in which the associated group clustered together is shown next to the branches from 1000 bootstrap replications. Primary branches which occur in less than 50% of trees were collapsed. The tree is drawn to scale, with branch lengths measured in the number of amino acid replacements per site. The analysis involved 155 amino acid sequences with a final alignment length of 64 positions. All positions containing gaps and missing data were eliminated.

File Name: Supplementary Data 3
Description: Molecular Phylogenetic analysis by Maximum Likelihood method of IL-1 family, IL-18, IL-33 and FGF proteins demonstrating that IL-33 and IL-18 appear to be as equally unrelated to the IL-1 family as the FGF family, demonstrating that IL-18 and IL-33 should not be included in the IL-1 ancestral family. The evolutionary history was inferred by using the Maximum Likelihood method based on the JTT matrix-based model. The tree with the highest log likelihood (-10427.12) is shown. The percentage of trees in which the associated taxa clustered together is shown next to the branches. The tree is drawn to scale, with branch lengths measured in the number of substitutions per site. The analysis involved 344 amino acid sequences. All positions containing gaps and missing data were eliminated. There were a total of 52 positions in the final dataset.

File Name: Supplementary Data 4
Description: Molecular Phylogenetic analysis by Maximum Likelihood method of HAX-1 proteins demonstrating that it is present across a wide range of clades suggesting it first appear prior to divergence of arthropod and vertebrates during the Cambrian period ~540 million years ago . The 4 evolutionary history was inferred by using the Maximum Likelihood method based on the JTT matrix-based model. The tree with the highest log likelihood (-1320.38) is shown. The percentage of trees in which the associated taxa clustered together is shown next to the branches. The tree is drawn to scale, with branch lengths measured in the number of substitutions per site. There were a total of 37 positions from 35 amino acid sequences in the final dataset.

File Name: Supplementary Data 5
Description: Molecular Phylogenetic analysis by Maximum Likelihood method of IL-1α proteins demonstrating that there is not an accelerated mutation rate in the toothed whale with similar evolutionary distances between toothed whales and baleen whales as other related mammalian clades. Indicating that the loss of the NLS in toothed whales did not lead to a significantly altered IL-1α sequence or subsequent function. This supports the notion that the key function driving IL-1α divergence from IL-1β and continued retention is not in the nucleus. The evolutionary history was inferred by using the Maximum Likelihood method based on the JTT matrix based model. The tree with the highest log likelihood (-2868.16) is shown. The percentage of trees in which the associated taxa clustered together is shown next to the branches. The tree is drawn to scale, with branch lengths measured in the number of substitutions per site. The analysis involved 18 amino acid

sequences. All positions containing gaps and missing data were eliminated. There were a total of 191 positions in the final dataset.

File Name: Supplementary Data 6
Description: Molecular Phylogenetic analysis by Maximum Likelihood method of IL-1 family subgroup proteins demonstrating greater stability and more sequence coverage compared to the IL-1 superfamily constructed tree. The evolutionary history was inferred by using the Maximum Likelihood method based on the JTT matrix-based model. The tree with the highest log likelihood (-6736.57) is shown. The percentage of trees in which the associated taxa clustered together is shown next to the branches. The tree is drawn to scale, with branch lengths measured in the number of substitutions per site. The analysis involved 116 amino acid sequences. All positions containing gaps and missing data were eliminated. There were a total of 70 positions in the final dataset.

File Name: Supplementary Data 7
Description: Molecular Phylogenetic analysis by Maximum Likelihood method of IL-18 proteins demonstrating greater stability and more sequence coverage compared to the IL-1 superfamily constructed tree. The evolutionary history was inferred by using the Maximum Likelihood method based on the JTT matrix-based model. The tree with the highest log likelihood (-3947.49) is shown. The percentage of trees in which the associated taxa clustered together is shown next to the branches. The tree is drawn to scale, with branch lengths measured in the number of substitutions per site. The analysis involved 18 amino acid sequences. All positions containing gaps and missing data were eliminated. There were a total of 146 positions in the final dataset. Evolutionary analyses were conducted in MEGA7.

File Name: Supplementary Data 8
Description: Molecular Phylogenetic analysis by Maximum Likelihood method of IL-33 proteins demonstrating greater stability and more sequence coverage compared to the IL-1 superfamily constructed tree. The evolutionary history was inferred by using the Maximum Likelihood method based on the JTT matrix-based model. The tree with the highest log likelihood (-2809.97) is shown. The percentage of trees in which the associated taxa clustered together is shown next to the branches. The tree is drawn to scale, with branch lengths measured in the number of substitutions per site. The analysis involved 14 amino acid sequences. All positions containing gaps and missing data were eliminated. There were a total of 233 positions in the final dataset.

File Name: Supplementary Data 9
Description: Gene IDs and sequence status of all genes used.
